# Association Between Dietary Inflammatory Index and Mental Health: A Systematic Review and Dose–Response Meta-Analysis

**DOI:** 10.3389/fnut.2021.662357

**Published:** 2021-05-05

**Authors:** Guo-Qiang Chen, Chun-Ling Peng, Ying Lian, Bo-Wen Wang, Peng-Yu Chen, Gang-Pu Wang

**Affiliations:** ^1^Shandong Engineering Laboratory for Health Management, Department of Health Management, Department of Medical Record Management and Statistics, The First Affiliated Hospital of Shandong First Medical University, Shandong Provincial Qianfoshan Hospital, Jinan, China; ^2^Department of Medical Record Management and Statistics, Shandong Provincial Qianfoshan Hospital, The First Affiliated Hospital of Shandong First Medical University, Jinan, China; ^3^Qilu Children's Hospital of Shandong University, Jinan, China; ^4^YouJiang Medical University for Nationalities, Baise, China; ^5^The Fourth People's Hospital of Jinan City, Jinan, China

**Keywords:** mental health, dietary inflammatory index, depression, anxiety, dose-response meta-analysis

## Abstract

**Objective:** We aimed to systematically evaluate the association between Dietary Inflammatory Index (DII) and mental health.

**Methods:** We searched PubMed, Embase, and Web of Science from their inception to December 31, 2020. Categorical meta-analysis and dose–response meta-analysis were performed to evaluate the association between DII and mental health.

**Results:** A total of 16 studies were included in this meta-analysis. Compared with the lowest DII category, the highest category was significantly associated with a variety of mental health outcomes, with the following pooled odds ratios (ORs) and 95% confidence intervals (95% CIs): 1.28 (1.17–1.39) for symptoms of depression, 1.27 (1.08–1.49) for symptoms of anxiety, 1.85 (1.43–2.40) for distress, and 4.27 (1.27–14.35) for schizophrenia. Furthermore, there was a linear dose–response relationship between DII and symptoms of depression in that a 1-unit increment in DII was associated with an increased risk of 6% for symptoms of depression (OR: 1.06, 95% CI: 1.03–1.19).

**Conclusion:** The present study indicates that more pro-inflammatory diet, as estimated by the higher DII score, is associated with symptoms of mental disorder. It may be of clinical and public health significance regarding the development of novel nutritional psychiatry approaches to promote good mental health.

## Introduction

Mental health disorders, as the leading cause of disability, represent a major public health concern (1, 2). It is estimated by WHO that one in four people worldwide is affected by mental health disorders in his or her lifetime, with around 450 million people currently suffering from such conditions (3). Considering the significant prevalence and associated socioeconomic burden, early identification of the modifiable factors consists crucial preventive strategies against the development of mental disorders and their progression to serious complications.

Among the modifiable factors, diet is one of the main lifestyle-related factors for mental disorders that individuals are exposed to daily. Of note, the experts from the International Society for Nutritional Psychiatry Research state that “diet and nutrition are central determinants of mental health” (4). It has been increasingly recognized that diet could serve as a key source of inflammation due to the ability of specific food parameters to regulate inflammatory biomarkers (5–7). Some specific nutrients with presumed pro-inflammatory properties, such as red meat, fried food, and high-fat dairy products, are associated with a higher likelihood of developing mental disorders (8–10). Meanwhile, existing systematic reviews have shown that healthy dietary patterns with presumed anti-inflammatory features, such as the Mediterranean diet characterized by high intakes of vegetables, fruit, fish, and healthy oils, are associated with a lower risk of mental disorders (11). Therefore, it is proposed that diet-induced inflammation may serve to be one of potential pathways through which diet links to mental health outcomes.

To better understand the inflammatory potential of diet, the Dietary Inflammatory Index (DII) was developed to assess the inflammatory capacity of the overall diet according to the pro- and anti-inflammatory efficacy of different dietary components (12), which has been validated successfully with various inflammatory markers (13, 14). Existing epidemiological studies have explored the association between DII and mental health disorders, with some reporting an increased risk associated with a higher DII, and others, no association (15, 16). Recent systematic reviews indicate that a higher DII is associated with an increased risk of depression; however, the strength and shape of the dose–response relationship have not been determined (17). Furthermore, although there is evidence to suggest that the biological mechanism underlying the association between DII and mental health is not just limited to depression, previous systematic reviews have only focused on the very particular aspect of mental health outcomes (18, 19), and no review has investigated the effect of DII on the other kinds of mental health symptoms or disorders, such as anxiety, distress, and schizophrenia.

The inconsistent findings of previous research and the lack of exhaustive overview on different mental health outcomes make it difficult to draw a reliable and universal conclusion. Therefore, the present meta-analysis was undertaken to provide an updated, comprehensive, and dose–response review about the association between DII and a broader range of mental health symptoms or disorders.

## Methods

We formulated research questions following the Population, Intervention, Comparator, Outcomes and Study Design (PICOS) strategy. In the form of PICOS, the study was described as follows: P, patients with mental health symptoms or disorders; I, patients with higher DII level; C, patients with lower DII level; O, mental health symptoms or disorders; S, cohort, case-control, or cross-sectional study. This systematic review was performed following the guidelines of the Preferred Reporting Items for Systematic Reviews and Meta-Analyses (PRISMA) (20). The PRISMA checklist was shown in Supplementary Table 1.

### Search Strategy

A comprehensive search was conducted to identify relevant articles in PubMed, Web of Science, and Embase from their inception to December 31, 2020. Search terms were as follows: (diet^*^) AND (inflammat^*^) AND (depress^*^ OR anxi^*^ OR emotion^*^ OR affect^*^ OR ^*^stress OR schizophrenia OR mental OR psychological OR psychiatric). In addition, the reference lists of all relative reviews and articles were also manually searched.

### Eligibility Criteria

Studies were included if they met the following inclusion criteria: (1) the study design was case-control, cohort, or cross-sectional study; (2) the DII was the exposure of interest; (3) the outcome of interest should be at least one kind of mental health symptom or disorder, as determined by a clinical diagnosis, or a validated self-report scale with a standardized cutoff point, including depression, anxiety, distress, and schizophrenia; (4) the study reported adjusted risk estimate with their corresponding 95% confidence interval (CI). If data were duplicated or shared in more than one study, the study with the largest dataset was included.

### Data Extraction and Quality Assessment

The following information was extracted from each included study: the first author's name, journal, year of publication, country where the study was performed, study design, sex, age range or mean age (years), sample size, number of cases, follow-up period (if applicable), diet assessment, comparison of DII score, mental health assessment, covariates adjusted for in the statistical analysis, as well as multivariable-adjusted risk estimate with 95% CI for each category of DII. Two authors (GQC and CLP) independently extracted variables from all eligible studies into a predesigned form. Any discrepancy was discussed and resolved by consensus with another author (GPW). Quality of cohort and case-control studies was assessed using the Newcastle–Ottawa Quality Assessment Scale (NOS) with the score ranging from 0 to 9 (21). Quality of cross-sectional studies was assessed using Agency for Healthcare Research and Quality (AHRQ) scale, which includes 11 items. An item was scored 0 for “No” or “Unclear” and 1 for “Yes” (22).

### Statistical Analyses

For categorical meta-analysis, odds ratios (ORs) and the corresponding 95% CIs were initially pooled for the highest vs. lowest category as well as the second highest vs. lowest category of DII. Cochran's *Q*-test and *I*^2^ were used to examine the heterogeneity among studies. *I*^2^ equaling 0–25% indicates that the heterogeneity might not be important; 25–50% represents moderate heterogeneity; 50–75% represents substantial heterogeneity; and 75–100% represents considerable heterogeneity. A fixed-effects model was used if no or low heterogeneity was detected; otherwise, the random-effects model was adopted. Subgroup analyses were conducted based on study design, gender, geographic location, and number of DII components according to an *a priori* protocol. Sensitivity analyses were conducted by excluding one study at one time from each analysis to confirm the robustness of our analyses. Publication bias was assessed by Egger's and Begg's tests. The trim-and-fill approach was performed to explore the adjusted effect size, taking publication bias into account.

Dose–response meta-analysis was conducted using the method developed by Greenland and Longnecker (23) and Orsini et al. (24). Studies with at least three quantitative categories of exposures were adopted. The median or mean of DII, cases, person-years or person, and risk estimate with 95% CI for each DII category of included studies were extracted for trend estimations. If the medians were not reported, we approximated it using the midpoint of upper and lower boundaries. If the upper boundary for the highest category was not provided, we assumed that the boundary had the same amplitude as the adjacent category. Potential non-linear relationships between DII and risk of mental disorders were examined by using restricted cubic splines, with 3 knots fixed at the 10th, 50th, and 90th percentiles of the distribution of DII. A *P*-value for non-linearity was calculated by testing the coefficient of the second spline equal to zero, as described previously (25). In addition, the two-stage generalized least squares regression was used to estimate the linear dose–response relationship for 1-unit increment in DII score with the risk of mental health disorders. All statistical analyses were performed using STATA version 14 (Stata Corporation, College Station, Texas, USA). *P*-values were considered significant at a level of <0.05.

## Results

### Study Characteristics

**Figure 1:**
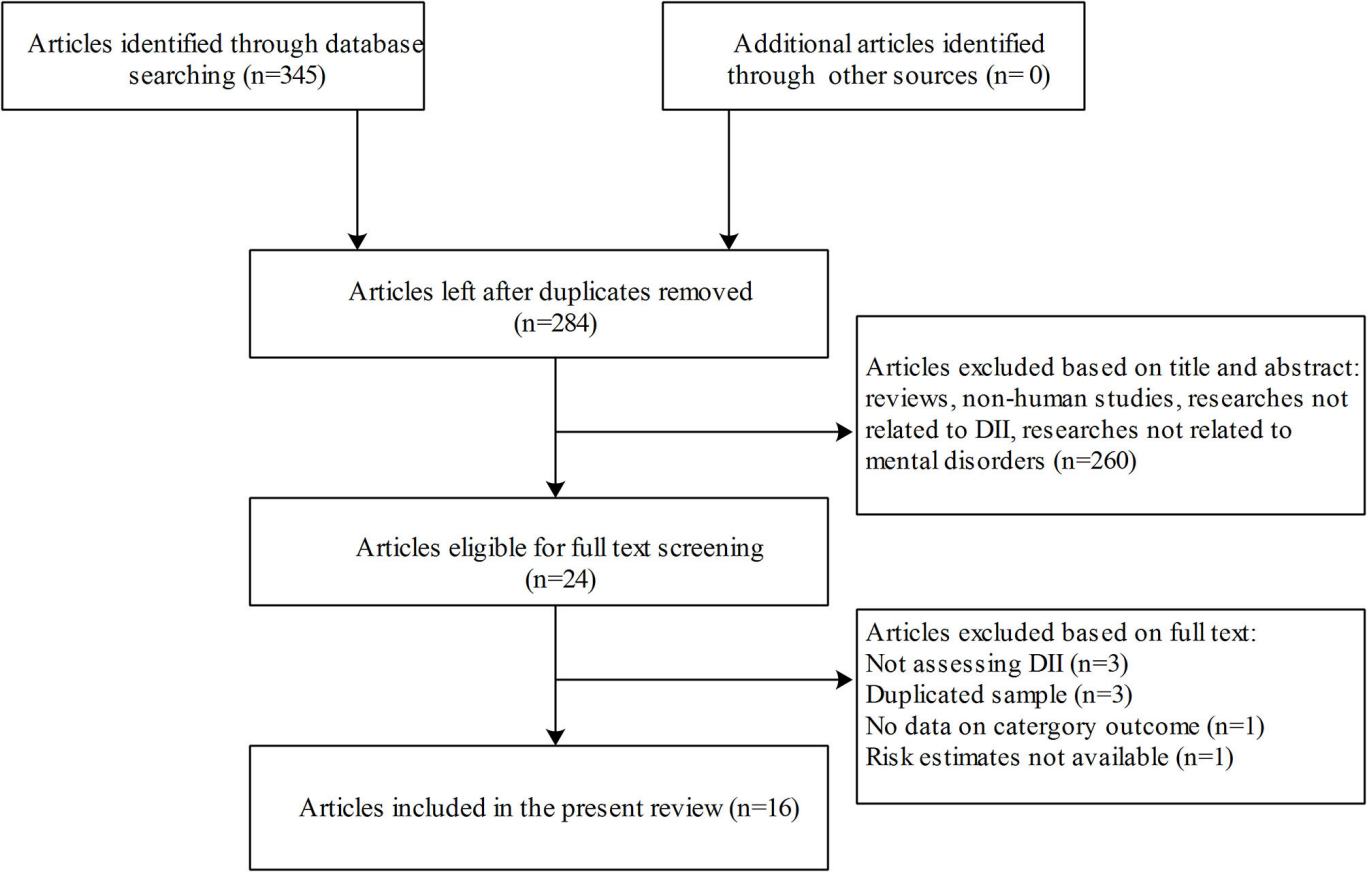
Flowchart for study selection process.

The process of literature selection is shown in Figure 1. A total of 16 articles with 92,242 participants were included in this meta-analysis, including five cohort studies, one case-control study, and 10 cross-sectional studies (16, 26–41). All studies assessed the DII score based on interviewed food-frequency questionnaires or 24-h diet recalls. Eight studies were conducted in Asia, four in American countries, three in Europe, and one in Australia. Of all the included studies, symptoms of depression were measured in 13 studies, symptoms of anxiety in four studies, distress in three studies, and schizophrenia in one study. The characteristics of all included studies are presented in Table 1. The mean quality score was 7.3 assessed by the NOS for cohort and case-control studies and 7.2 by the AHRQ for cross-sectional studies (Supplementary Tables S2a,b).

**Table 1:** The characteristics of studies included in the meta-analysis.

**References**	**Location**	**Case/Total No**.	**Sex: female**	**Age (mean /range)**	**Study design follow-up (years)**	**Measures of Outcome**	**Mental health assessment**	**Dietary assessment tool**	**DII score comparison**	**OR (95%CI)**	**Adjustment for covariates**
Sánchez-Villegas et al. (26)	Spain	1,051/15,093	58.70%	38.3	Cohort (8.5)	Depression	Self-reported physician provided diagnosis	FFQ	Quintile 5 vs. 1 Quintile 4 vs. 1 Quintile 3 vs. 1 Quintile 2 vs. 1	1.37 (1.09–1.73) 1.24 (1.00–1.53) 1.17 (0.95–1.43) 1.21 (0.99–1.47)	Age, sex, BMI, smoking, PA, vitamin supplements, TEI, presence of CVD, DM, hypertension or dyslipidemia
Shivappa et al. (27)	Australia	1,573/6,438	100%	52.0	Cohort (12)	Depressive symptoms	CES-D-10≥10	FFQ	Quartile 4 vs. 1 Quartile 3 vs. 1 Quartile 2 vs. 1	1.23 (1.05–1.45) 1.14 (0.97–1.32) 1.08 (0.93–1.25)	Total energy intake, highest qualification completed, marital status, menopause, night sweats, major personal illness or injury, lifestyle factors, smoking, PA, BMI, depression
Shivappa et al. (27)	USA	837/3,608	56.50%	61.4	Cohort (10)	Depressive symptoms	CES-D-20>20	FFQ	Quartile 4 vs. 1 Quartile 3 vs. 1 Quartile 2 vs. 1	1.24 (1.01–1.53) 1.06 (0.86–1.30) 1.21 (0.99–1.48)	Age; sex; race; body mass index; education; smoking habits; yearly income; Physical Activity Scale for Elderly score; Charlson Comorbidity Index; CES-D: Center for Epidemiologic Studies Depression Scale at baseline; statins use; NSAIDS or cortisone use
Adjibade et al. (28)	France	172/3,523	57.60%	52.1	Cohort (12.6)	Depressive symptoms	CES-D-10 scale≥17 for men and ≥23 for women	24-h diet recalls	Quartile 4 vs. 1 Quartile 3 vs. 1 Quartile 2 vs. 1	1.06 (0.66–1.71) 0.87 (0.55–1.39) 0.74 (0.47–1.18)	Age, sex, intervention group during the trial phase, education, energy intake, marital status, socio professional status, number of 24 h dietary records, interval between two CES-D measures.
Phillips et al., (16)	USA	NA/2,047	50.80%	50–69	Cross-sectional	Depressive symptoms Anxiety	CES-D-20 > 16 HADS>13	FFQ	Tertile 3 vs. 1 Tertile 2 vs. 1 Tertile 3 vs. 1 Tertile 2 vs. 1	1.36 (0.83–2.24) 1.69 (1.06–2.69) 1.38 (0.95–2.24) 1.33 (0.83–2.11)	Age and gender, BMI, physical activity, smoking and alcohol consumption, antidepressant use and history of depression.
Wirth et al. (38)	USA	1,648/18,875	50.70%	46.9	Cross-sectional	Depressive symptoms	PHQ-9≥10	24-h diet recalls	Quartile 4 vs. 1 Quartile 3 vs. 1 Quartile 2 vs. 1	1.13 (0.92–1.39) 1.14 (0.87–1.49) 1.08 (0.87–1.33)	Race, education, marital status, perceived health, current infection status, family history of smoking, smoking status, past cancer diagnosis, arthritis, age, and average nightly sleep duration.
Shivappa et al. (31)	Iran	43/300	100%	15–18	Cross-sectional	Depressive symptoms	DASS-21 > 9	FFQ	Tertile 3 vs. 1 Tertile 2 vs. 1	3.96 (1.12–13.97) 3.03 (1.11–8.26)	Age and energy, physical activity, BMI, smoking, presence of chronic disease, diet supplement use, salary and marital status
Açik et al. (33)	Turkey	79/134	100%	19–24	Cross-sectional	Depressive symptoms	Zung self-rating depression scale	24-h diet recalls	Tertile 3 vs. 1 Tertile 2 vs. 1	2.90 (1.51–5.98) 1.07 (0.48–2.48)	Age, smoking and alcohol consumption, physical activity, BMI, and energy intake
Shivappa et al. (34)	Iran	84/299	100%	15–18	Cross-sectional	Distress	DASS-21 >9	FFQ	Tertile 3 vs. 1 Tertile 2 vs. 1	3.48 (1.33–9.09) 3.16 (1.43–7.00)	Age, energy, physical activity, BMI, smoking, presence of chronic disease, diet supplement use, salary and marital status.
Bergmans et al. (30)	USA	1,486/11,592 2,089/11,592	52%	20–80	Cross-sectional	Distress Symptoms of anxiety	HRQOL HRQOL	24-h diet recalls	Quintile 5 vs. 1 Quintile 4 vs. 1 Quintile 3 vs. 1 Quintile 2 vs. 1 Quintile 5 vs. 1 Quintile 4 vs. 1 Quintile 3 vs. 1 Quintile 2 vs. 1	1.81 (1.2–2.71) 1.42 (0.95–2.11) 1.27 (0.90–1.80) 1.02 (0.72–1.46) 1.64 (1.14–2.35) 1.38 (1.02–1.88) 1.24 (0.95–1.62) 1.29 (0.99–1.68)	Age and gender, race/ethnicity, poverty income ratio category, employment status, health insurance status, educational status, and marital status, BMI, smoking, physical activity, sedentary time, use of vitamin supplements, total energy intake, menopause (among women), and any comorbidity.
Salari-Moghaddam et al., 2018	Iran	963/3,363 779/3,363 456/3,363	58.25%	36.3	Cross-sectional	Depressive symptoms Distress Symptoms of anxiety	HADS GHQ HADS	FFQ	Quintile 5 vs. 1 Quintile 4 vs. 1 Quintile 3 vs. 1 Quintile 2 vs. 1 Quintile 5 vs. 1 Quintile 4 vs. 1 Quintile 3 vs. 1 Quintile 2 vs. 1 Quintile 5 vs. 1 Quintile 4 vs. 1 Quintile 3 vs. 1 Quintile 2 vs. 1	1.84 (1.30–2.60) 1.70 (1.21–2.40) 1.49 (1.06–2.10) 1.17 (0.83–1.66) 1.72 (1.20–2.46) 1.44 (1.01–2.05) 1.18 (0.82–1.69) 1.04 (0.72–1.50) 1.69 (1.07–2.67) 1.34 (0.85–2.10) 1.26 (0.80–2.00) 0.96 (0.60–1.55)	Age, sex, energy intake, marital status, education, family size, home ownership, antidepressant use, vitamin supplements use, smoking status, physical activity, chronic conditions and BMI
Jahrami et al. (36)	Bahrain	120/240	54.17%	20–60	Case–Control	Schizophrenia	ICD-10	FFQ	Quartile 4 vs. 1 Quartile 3 vs. 1 Quartile 2 vs. 1	5.96 (1.74–20.38) 2.78 (0.77–10.0) 4.27 (1.27–14.35)	Age, sex, body mass index, education, employment, diabetes, hypertension, and cardiovascular disease
Adjibade et al. (35)	France	2,221/26,730	76.24%	18–86	Cohort (5.4)	Depressive symptoms	CES-D ≥17 for men ≥23 for women	24-h diet recalls	Quartile 4 vs. 1 Quartile 3 vs. 1 Quartile 2 vs. 1	1.16 (1.02–1.32) (0.89–1.15) 0.97 (0.86–1.10)	Age, sex, marital status, educational level, occupational categories, household income per consumption unit, residential area, energy intake without alcohol, number of 24 h-dietary records, and inclusion month, alcohol intake, smoking status, physical activity, and BMI, health events during follow-up (cancer, type 2 diabetes, and cardiovascular events).
Shin et al. (39)	Korea	752/15,929	54.79%	≥19	Cross-sectional	Depressive symptoms	PHQ score of ≥10		Tertile 3 vs. 1 Tertile 2 vs. 1	1.65 (1.14–2.39) 1.39 (0.98–1.96)	Age, gender, education, occupation, alcohol consumption, smoking status, physical activity, and BMI.
Ghazizadeh et al. (40)	Iran	2,631/7,083 3,580/7,083	57.5%	35–65	Cross-sectional	Depressive symptoms Symptoms of anxiety	BDI-II ≥14 BAI ≥ 7		Female Quartile 4 vs. 1 Quartile 3 vs. 1 Quartile 2 vs. 1 Male Quartile 4 vs. 1 Quartile 3 vs. 1 Quartile 2 vs. 1 Female Quartile 4 vs. 1 Quartile 3 vs. 1 Quartile 2 vs. 1 Male Quartile 4 vs. 1 Quartile 3 vs. 1 Quartile 2 vs. 1	1.18 (1.05–1.33) 1.10 (0.97–1.24) 0.97 (0.85–1.09) 1.17 (0.95–1.43) 0.98 (0.80–1.19) 0.85 (0.65–1.12) 1.09 (0.95–1.25) 1.03 (0.89–1.19) 1.08 (0.94–1.25) 1.18 (0.98–1.43) 1.03 (0.76–1.39) 1.08 (0.90–1.29)	Age, BMI, smoking status, education level, marital status, physical activity level, high sensitivity C-reactive protein, and dyslipidemia.
Moludi et al. (41)	Iran	275/4,630	100%	35–65	Cross-sectional	Depressive symptoms	Screening questionnaire	FFQ	Tertile 3 vs. 1 Tertile 2 vs. 1	1.47 (1.07–2.03) 1.27 (0.92–1.75)	Age, BMI, smoking, alcohol abuse, physical activity, and place of living.

*FFQ, Food frequency questionnaire; CES-D, Center for Epidemiologic Studies Depression Scale; HRQOL, Health-Related Quality of Life; GHQ, General Health Questionnaire; HADS, Hospital Anxiety and Depression Scale; PHQ-9, Patient Health Questionnaire-9; DASS, Depression Anxiety and Stress Scale; ICD-10, International Statistical Classification of Diseases-10; BDI-II, Beck Depression Inventory II; BAI, Beck Anxiety Inventory*.

### Association Between Dietary Inflammatory Index and Symptoms of Depression

Here, 13 studies (five cohort studies with 55,392 participants and eight cross-sectional studies with 52,361 participants) investigated the association between DII and symptoms of depression. A significant association was found between the highest DII category and symptoms of depression (pooled OR: 1.28, 95% CI: 1.17–1.39) compared with the lowest category, with moderate heterogeneity (*I*^2^ = 39.6%, *P* = 0.06). Sensitivity analyses showed that the pooled ORs and 95% CIs did not alter substantially by removing any one study, confirming the stability of the present results. Both Egger's and Begg's tests revealed significant publication bias, and the *P-*values were 0.01 and 0.01, respectively. After imputing four missing studies using the trim-and-fill method, the recalculated pooled OR did not substantially change from the initial estimate (imputed OR: 1.21, 95% CI: 1.14–1.27). The pooled OR of symptoms of depression was 1.15 (95% CI: 1. 05–1.25) for the second highest vs. lowest DII category, with moderate heterogeneity (*I*^2^ = 38.8%, *P* = 0.07). There was evidence of publication bias (*P* = 0.04 for Begg's test, *P* = 0.03 for Egger's test). After imputing four missing studies using the trim-and-fill method, the recalculated pooled OR did not substantially change from the initial estimate (imputed OR: 1.08, 95% CI: 1.02–1.15).

**Figure 2:**
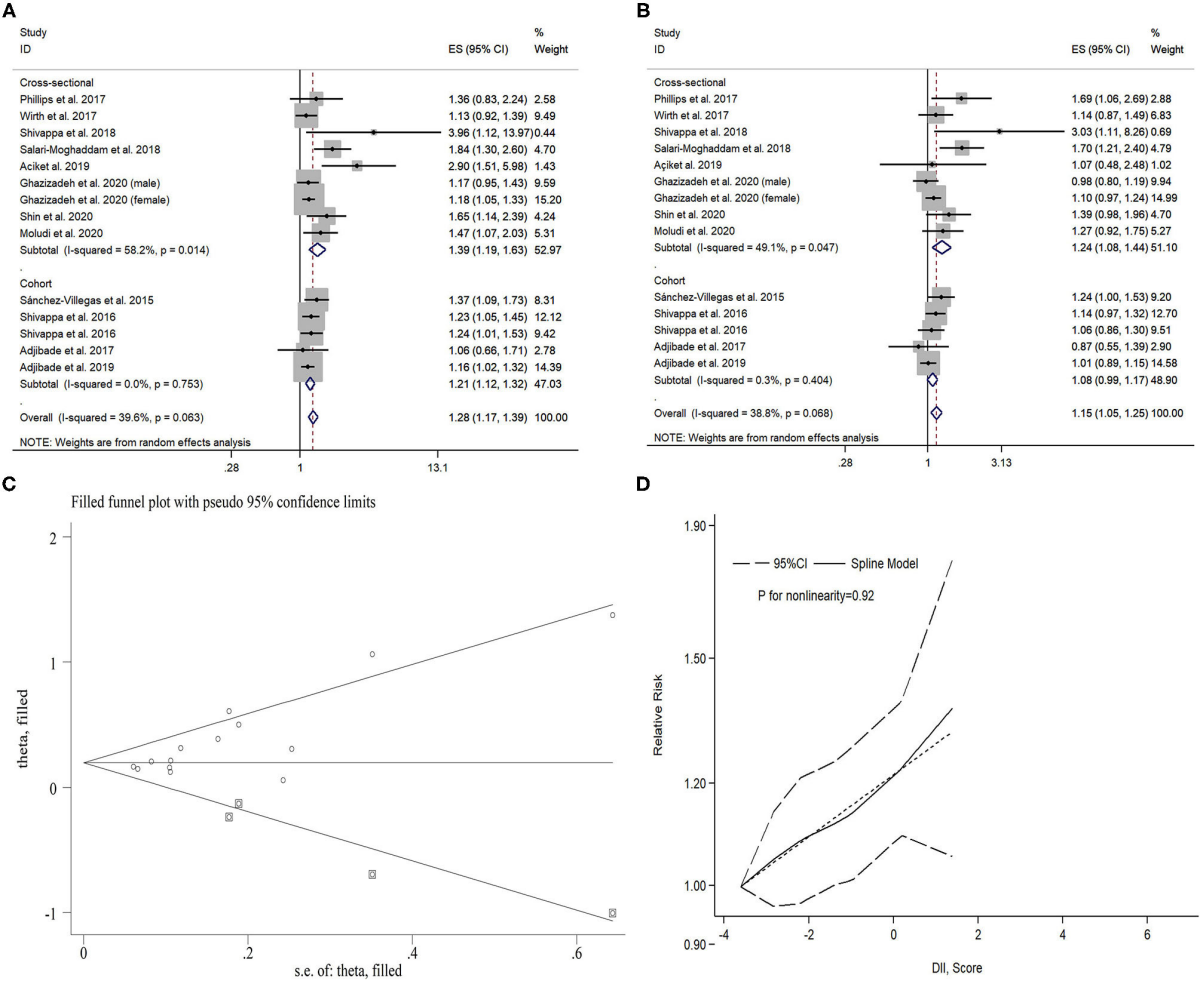
Forest plot of the pooled effect estimates of symptoms of depression. **(A)** The highest Dietary Inflammatory Index (DII) category compared with the lowest category. **(B)** The second highest DII category compared with the lowest category. **(C)** Filled funnel plot with 95% CI using the trim-and-fill method. **(D)** Dose–response relationship between DII and symptoms of depression.

For dose–response meta-analysis, it was shown that there was no significant non-linear relationship between DII and symptoms of depression (*P*_nonlinearity_ = 0.92). The pooled OR for 1-unit increment in DII was 1.06 (95% CI: 1.03–1.09) in linear dose–response analysis. More details can be seen in Table 2 and Figure 2.

**Table 2:** Results of subgroup analyses for DII and mental disorders.

	**The highest category**				**The second highest category**			
**Type of mental disorders**	**Studies, *n***	**OR (95% CI)**	***I^**2**^* (%)**	***P***	**Studies, *n***	**OR (95% CI)**	***I^**2**^* (%)**	***P***
**Symptoms of depression**								
All study	13	1.28 (1.17–1.39)	39.6	0.06	13	1.15 (1.05–1.25)	38.7	0.07
Study design								
Cohort study	5	1.21 (1.12–1.32)	0	0.75	5	1.08 (0.99–1.17)	0.3	0.40
Cross-sectional study	8	1.39 (1.19–1.63)	58.2	0.01	8	1.24 (1.08–1.44)	49.0	0.05
Gender								
Male	6	1.14 (0.99–1.31)	16.4	0.31	6	1.06 (0.93–1.22)	0	0.46
Female	10	1.34 (1.17–1.54)	56.9	0.01	10	1.31 (1.05–1.21)	30.6	0.16
Location								
America	3	1.20 (1.04–1.38)	0	0.72	3	1.18 (0.95–1.45)	37.9	0.20
Asia	6	1.50 (1.22–1.83)	66.3	<0.01	6	1.24 (1.04–1.47)	54.2	0.04
Europe	3	1.20 (1.07–1.34)	0	0.41	3	1.07 (0.90–1.26)	39.9	0.19
Australia	1	1.23 (1.05–1.45)	–	–	1	1.14 (0.97–1.32)	–	–
DII components								
<30	8	1.32 (1.19–1.46)	19.2	0.28	8	1.23 (1.11–1.36)	20.9	0.26
≥30	5	1.22 (1.06–1.42)	51.7	0.06	5	1.04 (0.94–1.15)	20.3	0.28
**Symptoms of anxiety**								
All study	4	1.27 (1.08–1.49)	45.4	0.12	4	1.11 (0.99–1.24)	10.0	0.35
Study design								
Cross-sectional study	4	1.27 (1.08–1.49)	45.4	0.12	4	1.11 (0.99–1.24)	10.0	0.35
Gender								
Male	3	1.23 (0.85–1.77)	29.4	0.24	3	1.26 (0.74–2.14)	50.7	0.13
Female	3	1.32 (0.94–1.86)	50.7	0.13	3	1.05 (0.92–1.20)	0	0.68
Location								
America	2	1.53 (1.16–2.01)	0	0.55	2	1.36 (1.06–1.76)	0	0.90
Asia	2	1.18 (1.00–1.38)	40.8	0.19	2	1.05 (0.93–1.19)	0	0.55
DII components								
<30	3	1.57 (1.24–1.99)	0	0.78	3	1.36 (1.09–1.70)	0	0.99
≥30	1	1.12 (1.00–1.25)	–	–	1	1.03 (0.90–1.17)	–	–
**Distress**								
All study	3	1.85 (1.43–2.40)	0	0.40	3	1.62 (1.14–2.31)	41.9	0.18
Study design								
Cross-sectional study	3	1.85 (1.43–2.40)	0	0.40	3	1.62 (1.14–2.31)	41.9	0.18
Gender								
Male	1	2.09 (1.09–4.02)	–	–	1	1.84 (0.92–3.66)	–	–
Female	1	1.61 (1.03–2.50)	–	–	1	1.34 (0.88–2.04)	–	–
Location								
America	1	1.81 (1.20–2.72)	–	–	1	1.42 (0.95–2.12)	–	–
Asia	2	2.11 (1.13–3.96)	44.8	0.18	2	1.96 (0.92–4.16)	68.1	0.08
DII components								
<30	2	1.76 (1.34–2.30)	0	0.85	2	1.43 (1.10–1.86)	0	0.96
≥30	1	3.48 (1.33–9.09)	–	–	1	3.16 (1.43–7.00)	–	–
**Schizophrenia**								
Case-control study	1	4.27 (1.27–14.35)	–	–		2.78 (0.77–10.00)	–	–

*FFQ: Food frequency questionnaire, CES-D: Centre for Epidemiologic Studies Depression Scale, HRQOL: Health-Related Quality of Life, GHQ: General Health Questionnaire, HADS: Hospital Anxiety and Depression Scale, PHQ-9: Patient Health Questionnaire-9, DASS: Depression Anxiety and Stress Scale, ICD-10: International Statistical Classification of Diseases-10, BDI-II: Beck Depression Inventory II, BAI: Beck Anxiety Inventory. DII, Dietary Inflammatory Index.*

### Association Between Dietary Inflammatory Index and Symptoms of Anxiety

**Figure 3:**
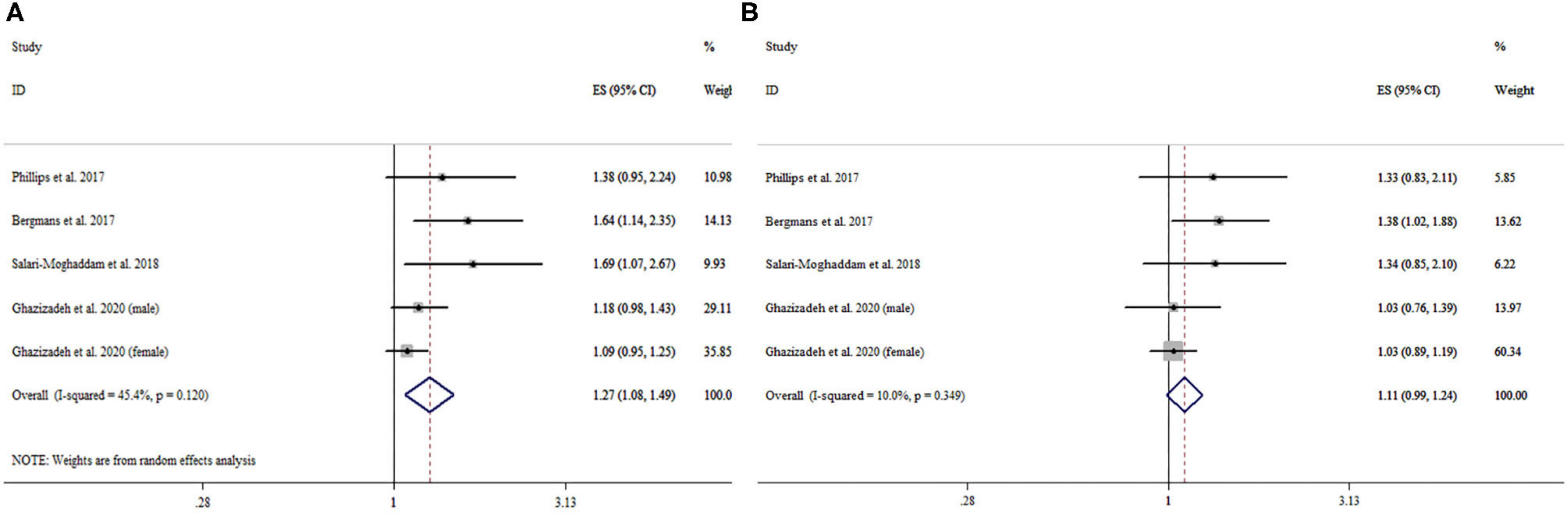
Forest plot of the pooled effect estimates of symptoms of anxiety. **(A)** The highest Dietary Inflammatory Index (DII) category compared with the lowest category. **(B)** The second highest DII category compared with the lowest category.

The association between DII and symptoms of anxiety was investigated in four cross-sectional studies, with a total of 21,632 participants. The pooled OR for the highest vs. lowest DII category was 1.27 (95% CI: 1.08–1.49), with moderate heterogeneity (*I*^2^ = 45.4%, *P* = 0.12). The pooled OR for the second highest vs. lowest DII category was 1.11 (95% CI: 0.99–1.24), with no significant heterogeneity (*I*^2^ = 10.0%, *P* = 0.35). More details can be seen in Table 2 and Figure 3.

### Association Between Dietary Inflammatory Index and Distress

**Figure 4:**
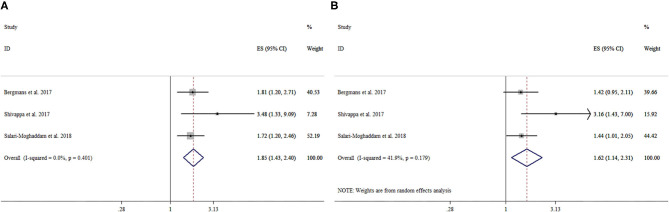
Forest plot of the pooled effect estimates of distress. **(A)** The highest Dietary Inflammatory Index (DII) category compared with the lowest category. **(B)** The second highest DII category compared with the lowest category.

There were three cross-sectional studies with a total of 15,254 participants investigating the association between DII and distress. The pooled OR for the highest vs. lowest DII category was 1.85 (95% CI: 1.43–2.40), with no significant heterogeneity (*I*^2^ = 0%, *P* = 0.40). The pooled OR for the second highest vs. lowest DII category was 1.62 (95% CI: 1.14–2.31), with moderate heterogeneity (*I*^2^ = 41.9%, *P* = 0.18). More details can be seen in Table 2 and Figure 4.

### Association Between Dietary Inflammatory Index and Schizophrenia

Only one study reported the association between DII and schizophrenia. The OR (95% CIs) of schizophrenia were 4.27 (1.27–14.35) and 2.78 (0.77–10.00) for the highest and second highest categories compared with the lowest DII category.

## Discussion

This systematic review and meta-analysis provided a comprehensive evaluation of current evidence on the association between DII and a great variety of mental health outcomes. The findings indicated that higher DII was associated with an increased risk of common mental health outcomes, including symptoms of depression, symptoms of anxiety, distress, as well as schizophrenia. Particularly important, there is a novel conclusion from dose–response analysis that 1-unit increment of DII was associated with a 6% higher risk of depressive symptoms.

Our findings indicated a significant association between pro-inflammatory diet and depression, which is in line with evidence from a recent meta-analysis. However, a previous meta-analysis on this topic did not perform subgroup analyses, sensitivity analyses, and publication bias test (17). A meta-analysis did not distinguish pro-inflammatory diet from unhealth dietary pattern (11), and another assessed the dietary inflammatory potential combining dietary and biomarker together (18). All above may potentially affect, to a certain degree, the precision and stability of pooled results. Specifically, our study presented a more comprehensive and clear understanding of the association between DII and depressive symptoms by performing a dose–response analysis and assessing dietary inflammatory potential through a simple and intuitive method. Importantly, we expanded on the previously described diet–depression association and suggested the potential implications of pro-inflammatory diet in a broad range of mental health outcomes, further reinforcing the role of diet in the pathophysiology of mental health symptoms or disorders.

The DII is a literature-derived, population-based diet quality index designed to standardize the inflammatory potential of an individual's diet (12). Up until the development of DII, there are two other categories of dietary indices used to clarify the association between diet and mental health outcomes. One category of these indices is derived using statistical methods (42), which closely matches the dietary habits of the studied population but does not necessarily reflect an optimal diet and is hardly replicable to other populations. Another category is developed based on healthy dietary guidelines, such as the Healthy Eating Index (HEI) (43), all of which do not target specific mechanisms. DII represents a unique biological mechanism underlying the diet–mental health link over other diet indices by capturing the inflammatory effect of diet. In addition, previous studies have demonstrated the predictive value of DII in chronic inflammatory disease, including obesity (44), cardiovascular disease (45), metabolic syndrome (46), and various types of cancers (47). All findings mentioned above indicate that the DII may potentially serve to be prevention targets of mental health disorders.

Depression, anxiety, and other common mental health symptoms or disorders have a high comorbidity, and it is well-documented that these disorders share genetic determinants as well as underlying neurobiological mechanisms (48, 49). Several potential mechanisms have been proposed to explain the observed association. First, the pro-inflammatory diet is associated with high levels of circulating inflammatory markers (50, 51). It has been shown that inflammatory markers, such as cytokines, could regulate neurotransmitter metabolism and neural plasticity, which in turn induce the development of neuropsychiatric diseases (52). Second, oxidative stress is implicated as an important determinant relevant to mental health disorders (53). It has been indicated that pro-inflammatory diet can modulate oxidative processes, and oxidant–antioxidant imbalance is associated with elevated levels of reactive oxygen and nitrogen species, which increase DNA damage (54). Such damage may underlie the demonstrated association between DII and mental health (54). In addition, the microbiome–gut–brain axis may represent a critical pathway through which a pro-inflammatory diet contributes to the etiology of mental disorders (55). It is demonstrated that pro-inflammatory diet can modify the gut microbiota composition and activity (56), and gut microbiota can potentially influence immune system activation, production of neurotransmitters, and regulation of neuroendocrine pathways, which in turn influence mental health (57, 58). Although the common mental health symptoms or disorders share mechanisms, the distinct pathophysiologic mechanisms for different disorders should be further elucidated in order to determine whether nutritional factors affect the development of these disorders differently.

A major strength of this study is that the meta-analysis provided a comprehensive overview on a wide range of mental health outcomes rather than a specific type related to DII, which provides convincing support of the diet–mental health link. Second, compared with previous studies on this topic, the current linear dose–response analysis can help clarify how the risk of depression changes along with the increase of the dietary inflammatory potential. Third, sensitivity analyses and detailed subgroup analyses further support the stability of our conclusions. Despite the strengths of the current systematic review, there are certain limitations that need to be addressed. First, cross-sectional design was used in most of the included studies, which did not give a causal relationship. Previous studies indicated that mental stress can lead to increased intake of high-energy and high-fat foods and result in a higher DII score, and it is probable that the bidirectional relationship exists between DII and mental health symptoms or disorders. Thus, well-designed cohort studies and randomized controlled trials are needed to further demonstrate the causal relationships. Second, although all original studies adjusted for different covariates, due to confounding biases inherent in each study, the possibility of remaining residual confounding is to be expected. Third, the results of this study might be affected by the moderate level of heterogeneity. Meta-regression analyses were used to explore the source of heterogeneity. The following independent variables including location and number of DII components were introduced into the meta-regression model. Finally, publication bias was observed in Begg's or Egger's tests, but using the trim-and-fill method to include supposedly missing negative studies, a significant association still persists. These limitations may impose a modest constraint on the interpretation of these findings, but they should not substantively undermine the internal validity of the study.

Our findings have significant implications for both public health and clinical practice. From the public health perspective, avoiding a pre-inflammatory diet could be a feasible approach in the primary prevention of adverse mental health. From the clinical perspective, the demonstrated associations may have potential benefits in formulating appropriate targeted therapeutic and intervention strategies for mental health symptoms or disorders. Therefore, future nutritional psychiatry research should aim to develop targeted nutritional protocols and then incorporate them into prevention and treatment guidelines of mental health symptoms or disorders.

In conclusion, more pro-inflammatory diet, as estimated by the higher DII score, could increase the risk of a variety of mental health disorders. It may be of public health and clinical significance regarding the development of novel nutritional psychiatry approaches to promote good mental health. Further well-designed prospective trials are needed to strengthen the evidence of the associations between the DII and mental health symptoms or disorders.

## Data Availability Statement

The data used and analyzed during the current study are available from the corresponding author on reasonable request.

## Author Contributions

G-QC and YL searched the databases, performed screening of titles and abstracts, performed screening of full texts, extracted data, performed all analyses, and wrote the manuscript. P-YC, B-WW, and C-LP were involved in revising the paper. G-PW supervised the study and contributed to the critical revision. All authors approved the final version of the manuscript.

## Conflict of Interest

The authors declare that the research was conducted in the absence of any commercial or financial relationships that could be construed as a potential conflict of interest.

## Supplementary Material

Supplementary Table 1 PRISMA Checklist.

Supplementary Table 2 Methodological quality of the included studies.
