# Supplementary material for: Association Between Dietary Inflammatory Index and Mental Health: A Systematic Review and Dose–Response Meta-Analysis
Source: Front Nutr. 2021 May 5;8:662357. doi: 10.3389/fnut.2021.662357 (PMC8133218; doi:10.3389/fnut.2021.662357)
Supplement: Supplementary Table 2 — Methodological quality of the included studies. [file Table_2.docx]

| Case-control study | Case definition | Representativeness | Control selection | Control definition | Comparability | Ascertainment of exposure | Same method of ascertainment for cases and controls | Non-Response rate |
| --- | --- | --- | --- | --- | --- | --- | --- | --- |
| Cohort study | Representativeness of the exposed cohort | Selection of the non-exposed cohort | Ascertainment of exposure | Demonstration that outcome of interest was not present at start of study | Comparability | Assessment of outcome | Follow-up long enough for outcomes to occur | Adequacy of follow up |
| Sánchez-Villegas  et al. 2015 | 0 | 1 | 1 | 1 | 2 | 1 | 1 | 0 |
| Shivappa et al. 2016 | 1 | 1 | 1 | 1 | 2 | 1 | 1 | 0 |
| Adjibade et al. 2017 | 0 | 1 | 1 | 1 | 2 | 1 | 1 | 0 |
| Shivappa et al. 2016 | 0 | 1 | 1 | 1 | 2 | 1 | 1 | 1 |
| [Jahrami et al. 2019](http://nc.yuntsg.com/pubmed/?term=Jahrami%20H%5BAuthor%5D&cauthor=true&cauthor_uid=31405205) | 1 | 1 | 0 | 1 | 2 | 1 | 1 | 0 |
| Adjibade et al. 2019 | 0 | 1 | 1 | 1 | 2 | 1 | 1 | 0 |

Table s2 a Methodological quality of included cohort and case-control studies in the meta-analysis

Table s2 b Methodological quality of included cross-sectional studies in the meta-analysis

| Item/Study | Define the source of information | List inclusion and exclusion criteria for exposed and unexposed subjects or refer to previous publications | Indicate time period used for identifying patients | Indicate whether or not subjects were consecutive if not population-based | Indicate if evaluators of subjective components of study were masked to other aspects of the status of the participants | Describe any assessments undertaken for quality assurance purposes | Explain any patient exclusions from analysis | Describe how confounding was assessed and/or controlled | If applicable, explain how missing data were handled in the analysis | Summarize patient response rates and completeness of data collection | Clarify what follow-up was expected and the percentage of patients for which incomplete data or follow-up was obtained |
| --- | --- | --- | --- | --- | --- | --- | --- | --- | --- | --- | --- |
| Phillips et al. 2017 | 1 | 1 | 1 | 1 | 0 | 0 | 1 | 1 | 0 | 1 | 0 |
| Wirth et al. 2017 | 1 | 1 | 1 | 1 | 0 | 0 | 1 | 1 | 1 | 1 | 0 |
| Bergmans et al. 2017 | 1 | 1 | 1 | 1 | 0 | 0 | 1 | 1 | 1 | 1 | 0 |
| Shivappa et al. 2018 | 1 | 1 | 1 | 1 | 0 | 0 | 1 | 1 | 1 | 1 | 0 |
| Salari-Moghaddam et al. 2018 | 1 | 1 | 1 | 1 | 0 | 0 | 1 | 1 | 1 | 1 | 0 |
| Aciket al. 2019 | 1 | 1 | 1 | 1 | 0 | 0 | 1 | 1 | 0 | 1 | 0 |
| Shivappa et al. 2017 | 1 | 1 | 0 | 1 | 0 | 0 | 1 | 1 | 0 | 0 | 0 |
| Ghazizadeh et al. 2020 | 1 | 1 | 1 | 1 | 0 | 0 | 1 | 1 | 0 | 1 | 0 |
| Shin et al. 2020 | 1 | 1 | 1 | 1 | 0 | 0 | 1 | 1 | 0 | 1 | 0 |
| Moludi et al. 2020 | 1 | 1 | 1 | 1 | 0 | 0 | 1 | 1 | 0 | 1 | 0 |
